# Effects of acute cervical stretching on arterial wall elastic properties

**DOI:** 10.3389/fphys.2023.1198152

**Published:** 2023-06-29

**Authors:** Harumi Ikebe, Naoya Oi, Akitoshi Makino, Daisuke Kume, Minenori Ishido, Tomohiro Nakamura, Masato Nishiwaki

**Affiliations:** ^1^ Graduate Course in Applied Chemistry, Environmental and Biomedical Engineering, Osaka Institute of Technology, Osaka, Japan; ^2^ Faculty of Human Studies, Taisei Gakuin University, Osaka, Japan; ^3^ Faculty of Engineering, Osaka Institute of Technology, Osaka, Japan; ^4^ Faculty of Information Science and Technology, Osaka Institute of Technology, Osaka, Japan

**Keywords:** arterial compliance, arterial stiffness, arteriosclerosis, exercise, flexibility, shear wave elastography

## Abstract

**Purpose:** Acute (immediate) or regular (mid- or long-term) stretching increases arterial compliance and reduces arterial stiffness. Stretching is widely known to induce arterial functional factor changes, but it is unclear whether stretching alters arterial structural factors. Ultrasound shear wave elastography can quantify the distribution of tissue elastic properties as an index of arterial structural factors. This study thus aimed to examine the effects of acute cervical stretching on arterial wall tissue elastic properties.

**Methods:** Seventeen healthy young adults participated in two different trials for 15 min in random order on separate days: a resting and sitting trial (CON) and a supervised cervical stretching trial (CS). In CS, subjects performed 10 different stretches. At each site, the stretch was held for 30 s followed by a 10-s relaxation period. In CON, subjects rested on a chair for 15 min.

**Results:** After the experiment, carotid arterial compliance, assessed by combined ultrasound imaging and applanation tonometry, was significantly increased in CS, but not in CON. However, there was no significant change in tissue elasticity properties of the arterial wall in either trial, as assessed by ultrasound shear wave elastography.

**Conclusion:** Acute cervical stretching significantly increased carotid artery compliance in young participants, but did not reduce elastic tissue properties (*i.e.*, arterial structural factors) of the carotid artery wall. These results strongly suggest that changes in structural factors have little relation to stretching-induced acute increases in arterial compliance.

## Introduction

Large arteries in the cardiothoracic region (*i.e.*, the central arteries) buffer the pulsation of systolic pressure and convert pulsatile cardiac ejection into continuous blood flow to the capillary beds (Tanaka, 2019). Arterial compliance and pulse wave velocity (PWV) reflect the status of arterial function for dilation and recoil (*i.e.*, buffering capacity), and have been widely accepted as indices of arterial distensibility and arterial stiffness (Arnett et al., 1994; Tanaka et al., 2000; Vlachopoulos et al., 2010; Tanaka, 2019). Because central arterial stiffening and a reduction in distensibility occur with aging, reductions in buffering capacity can increase systolic blood pressure (BP) and left ventricular hypertrophy and reduce continuous blood flow (O'Rourke, 1990; Arnett et al., 1994; Tanaka et al., 1998; O'Rourke and Hashimoto, 2007). In particular, decreases in carotid arterial distensibility and increases in central arterial stiffness are significantly associated with the risk of developing cardiovascular diseases and events as well as with mortality, as their function declines with age (Vlachopoulos et al., 2010; Ben-Shlomo et al., 2014; Kim et al., 2019). Thus, prevention or improvement of a reduction in distensibility and central arterial stiffening is very important for reducing the risk of cardiovascular disease, regardless of age.

Previous cross-sectional studies have reported a relationship between body flexibility and the status of arterial function (Yamamoto et al., 2009; Nishiwaki et al., 2014). This relationship is independent of BP, which is a major confounding factor (Nishiwaki et al., 2014). Furthermore, regular (*i.e.,* mid- or long-term) stretching exercises increase arterial compliance and/or reduce arterial stiffness (Cortez-Cooper et al., 2008; Nishiwaki et al., 2015; Shinno et al., 2017; Ikebe et al., 2022a). Acute (*i.e.,* immediate) local stretching exercises would also reduce corresponding local arterial stiffness (Yamato et al., 2017; Higaki et al., 2021; Ikebe et al., 2022b). Thus, these findings indicate that some physiological mechanisms (namely, arterial structural factors and/or arterial functional factors, but not confounding factors) participate in the relationship between body flexibility and arterial function status and stretching training adaptations. While these points remain controversial, recent studies have demonstrated that the arterial functional factors of blood flow and nitric oxide contribute to responses and adaptations to stretching exercise (Hotta et al., 2018; Bisconti et al., 2020; Yamato et al., 2021). However, whether stretching exercises *per se* can affect arterial structural factors is unclear, and these issues have not been addressed, especially in human studies.

Ultrasound shear wave elastography (SWE) is used as a noninvasive method for the quantitative evaluation of elastic change in tissues (Bamber et al., 2013). It uses a focused ultrasonic beam that emits shear waves into tissue and quantitatively evaluates the shear modulus from the resulting tissue shear rate. The stiffer the tissue, the faster the shear wave propagation velocity, which can be used as a noninvasive proxy for tissue stiffness (Bercoff et al., 2004; Palmeri et al., 2008). Previous studies have reported quantifiable changes in skeletal muscle stiffness with stretching (Hirata et al., 2017; Nakamura et al., 2017; Taljanovic et al., 2017). Interestingly, recent attempts have been made to assess arterial plaque and stiffness using SWE (Ramnarine et al., 2014; Garrard et al., 2015; Pruijssen et al., 2020; Thomas et al., 2020; Al-Mutairi et al., 2022). The measurement validity of the methods used to evaluate the arterial wall has been verified by phantoms in mechanical testing, *ex vivo*, and systematic review approaches (Maksuti et al., 2016; Widman et al., 2016; Pruijssen et al., 2020). A previous study has shown that the SWE method can quantify changes in carotid arterial wall stiffness with head movement (Thomas et al., 2020). A systematic review has also shown that SWE could detect statistically significant elasticity differences in patient/subject characteristics and could distinguish different plaque types with good reproducibility. These reports thus indicate that SWE can be used to assess the tissue elastic properties of the arterial wall *per se*.

Arteries have a three-layered structure consisting of tunica adventitia, tunica media, and tunica intima. The elements of the arterial wall are composed mainly of elastin, collagen, and vascular smooth muscle (Lee and Oh, 2010). Many studies have mentioned that biochemical changes in elastin-collagen composition (*i.e.*, structural determinants) seem unlikely to occur in a relatively short period (less than 1 year) (Tanaka et al., 2000; Nosaka et al., 2003; Maeda et al., 2005). However, direct stretch exercise stimulus may induce stretching of collagen fibers and acutely modulate their cross-linking in the local arterial wall, in addition to increasing pulse pressures. Thus, such changes in arterial structural factors can affect the elastic properties of tissue, thereby inducing an increase in arterial compliance and a reduction in arterial stiffness. Therefore, use of the SWE method to assess the tissue elastic properties of the arterial wall after stretching might enable detection of changes in arterial structural factors. The effects of stretching on arterial structural factors involved in the intrinsic tissue elastic properties of the artery are important in bridging the knowledge gap regarding arterial adaptation mechanisms to stretching and exercise; however, as far as we can ascertain, these have not yet been clarified.

Given this background, the aim of the present study was to test the hypotheses that cervical stretching would increase carotid arterial compliance and decrease tissue elastic properties of the carotid arterial wall, by an acute design and approach as an initial pilot study.

## Materials and methods

### Participants

The participants were 17 healthy young Japanese adults (male 13, female 4) who were recruited at our university. Mean age, height, body mass, body mass index (BMI), and body fat were 23.7 ± 1.3 years, 166.7 ± 1.8 cm, 63.5 ± 2.9 kg, 22.7 ± 0.8 kg/m^2^, and 23.3 ± 1.0%, respectively. None had any history of chronic disease that could affect cardiovascular health or metabolism. All participants were non-smokers and were not presently taking any medication and supplement. The purpose, procedures, and risks of the study were explained to all participants and written informed consent was obtained before enrolment in the study. All study protocols were reviewed and approved by the Human Ethics Committee at Osaka Institute of Technology (approval no. 2021-35) and were conducted in accordance with the tenets of the Declaration of Helsinki.

### Sample size and experimental procedures

We determined the appropriate and minimum sample size before the study by power calculations using G*Power 3.1 (Dusseldorf, Germany). A total sample size of at least 32 (sample size of 16 per trial) was needed to detect effect size (ES) (f) of 0.25 (medium) at 80% power with α of 5% using a within between interaction of two-way repeated measures analysis of variance (ANOVA). We further assumed that an alteration in arterial compliance would be shown transiently by a difference of ≥30% [ES (dz) of 0.8] in a pretest-posttest design, according to previous findings (Ikebe et al., 2022b). At least 15 participants were needed to detect this difference at 80% power with a two-tailed α of 5%. Therefore, we planned to recruit 17 participants (34 total sample size) in this study.

All experiments were conducted in a quiet, air-conditioned room at a temperature of 22°C–24°C during the daytime, and at the same number of hours after the last meal, to avoid potential diurnal variation. The start time of the trials was in the range 9:00 a.m.–17:00 p.m. The participants abstained from drinking beverages containing alcohol or caffeine and avoided strenuous physical activity for at least 24 h before participating in the experiment. On the day of the experiment, participants were advised to eat their habitual breakfast, lunch, and dinner at their usual mealtimes, and compliance was confirmed in terms of content and regularity by a checklist questionnaire and in face-to-face interviews.

**FIGURE 1:**
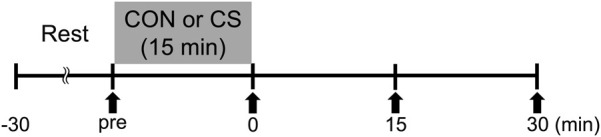
Experimental protocol. CON, control sitting trial; CS, cervical stretching trial; solid arrows, time points of measurement.

Figure 1 shows the time course of the study. Participants were assigned in random sequence to one trial per day for 2 days. The experimental trials comprised a resting and sitting trial as a control trial (CON), and a cervical stretching trial (CS). The trials were performed at least 1 day apart. Prior to each trial, participants arrived at the laboratory and rested for at least 30 min. Carotid arterial compliance, elastic tissue properties of the carotid arterial wall, systemic arterial stiffness, and hemodynamic parameters were then assessed in the supine position to establish pretrial baselines.

**FIGURE 2:**
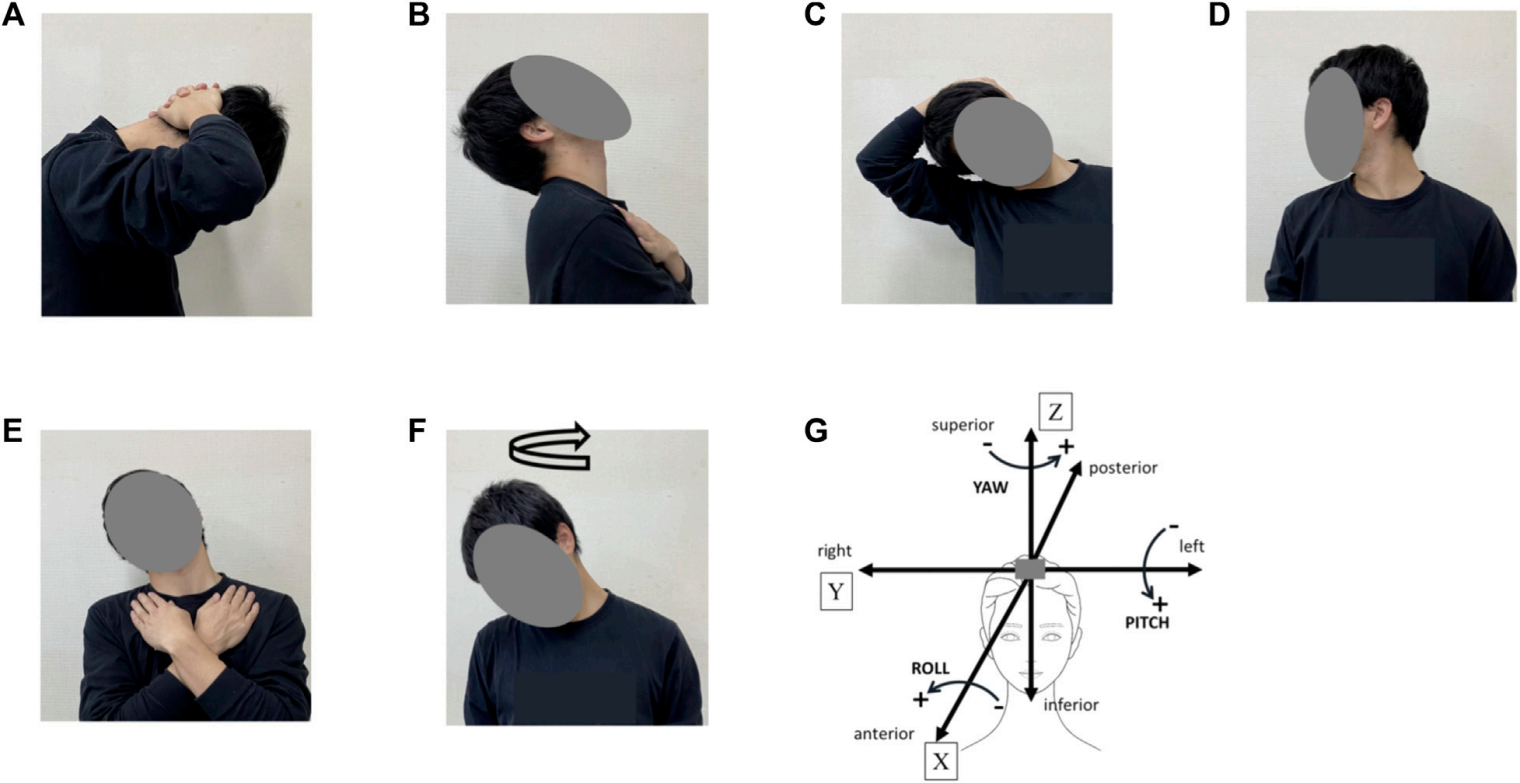
Cervical stretching program. **(A)**, cervical flexion; **(B)**, cervical extension; **(C)**, cervical lateroflexion (right), **(D)**, cervical rotation (right); **(E)**, cervical lateroflexion and extension (right); **(F)**, head rotation (turn right); **(G)**, definitions of head movement axis during cervical stretching. X indicates the anterior–posterior axis [sagittal axis] [“Roll,” left (−) and right (+)], Y indicates the left–right axis [frontal axis] [“Pitch,” forward (+) and backward (−)], and Z indicates the superior–inferior axis [vertical axis] [“Yaw”, left (+) and right (−)].

In the CON trial, subjects rested on a chair for 15 min. In the CS trial, participants performed the following 10 cervical stretching exercises in a 15 min sequence: flexion, extension, left and right lateroflexion, left and right rotation, left and right lateroflexion and extension, and left and right head rotation (Figure 2). For each exercise, self-stretching was held for 30 s at the end range (point of minimal discomfort) followed by a 10 s relaxation period, and this sequence was repeated twice during the 15 min trial. Because our previous report shows that direct trunk stretching reduces central arterial stiffness and increases carotid arterial compliance (*i.e.*, approximately corresponding local changes) (Ikebe et al., 2022b), static stretching of the cervical part was used as a method to alter carotid arterial compliance. To achieve targeted stretching of the carotid region, the cervical stretches were designed by physical therapists, and joint ranges of motion during the stretches were preliminarily confirmed using inertial sensors (Table 1). Heart rate (HR) was continuously monitored during each trial using a V800 heart rate monitor (Polar Japan, Tokyo, Japan), and averaged in 5 min periods. After each trial, the same measurements as for the pretrial baselines were repeated immediately (0–5 min), 15 min, and 30 min as a posttest.

**TABLE 1:** Joint angles during cervical stretching in the preliminary experiment.

Movement	X-axis			Y-axis			Z-axis		
Cervical flexion, deg		–		54	±	5		–	
Cervical extension, deg		–		−42	±	1		–	
Cervical right lateroflexion, deg	46	±	6		–			–	
Cervical left lateroflexion, deg	−42	±	10		–			–	
Cervical right rotation, deg		–			–		−47	±	8
Cervical left rotation, deg		–			–		74	±	8
Cervical right lateroflexion and extension, deg	17	±	4	−45	±	8		–	
Cervical left lateroflexion and extension, deg	−24	±	12	−31	±	11		–	
Right head rotation, deg/s (s/360°)	60 ± 13 (5 ± 1)								
Left head rotation, deg/s (s/360°)	56 ± 12 (6 ± 1)								

Joint angles of cervical and head parts were assessed by using wearable inertial sensors (Xsens DOT, Xsens (Movella), Enschede, Netherlands) during the cervical stretching (*n* = 3). Two sensors were placed on the head and chest, and data during each 30 s stretching were recorded with a sampling frequency of 60 Hz. Obtained acceleration data were processed by butterworth lowpass filtering using customized software, and then joint angles of cervical and head parts were calculated. X, Y, and Z shows the anterior-posterior axis [sagittal axis] [“Roll,” left (−) and right (+)], the left-right axis [frontal axis] [“Pitch,” forward (+) and backward (−)], and the superior-inferior axis [vertical axis] [“Yaw,” left (+) and right (−)], respectively.

Data are expressed as mean ± SEM.

### Assessment of parameters

The same investigators measured all parameters. Carotid arterial compliance was determined using a combination of ultrasound imaging of the common carotid artery diameter and applanation tonometry recording of the carotid artery in the supine position (Tanaka et al., 2000). A longitudinal image of the common carotid artery was measured using an ultrasound imaging system (Aixplorer; Supersonic Imagine, Aix-en-Provence, France) by the same experienced observer. A B-mode scan was obtained using a high-resolution multifrequency linear-array transducer (SL15-4; Supersonic Imagine). Longitudinal images of the common carotid artery were acquired for 10 successive beats at maximal (systolic) and minimal (diastolic) diameters. Arterial lumen diameter was measured offline using commercially available software (T.K.K. 5814; Takeikiki, Niigata, Japan). The pressure waveform and amplitude were obtained from the common carotid artery using a non-invasive pulse wave tonometer (SPT-301, Millar, Houston, United States) (Kelly et al., 1989; Tanaka et al., 2000; Miyachi et al., 2004). The pressure waveform was sampled at a frequency of 1,000 Hz through an analog-to-digital converter (Power Lab 2/26; AD Instruments Japan Inc., Nagoya, Japan) interfaced with a personal computer equipped with data acquisition software (Lab Chart 8; AD Instruments). Because the baseline levels of carotid blood pressure are subject to hold-down force, the pressure signal obtained by tonometry was calibrated by equilibrating the carotid mean arterial and diastolic blood pressures to the brachial artery values, as previously described (Armentano et al., 1995; Tanaka et al., 2000; Miyachi et al., 2004). Finally, arterial compliance was calculated using the equation [(D1−D0)/D0]/[2(P1−P0)] × π × D0^2^, where D1 and D0 are maximal and minimum arterial diameter, and P1 and P0 are the highest and lowest carotid BP, respectively (Tanaka et al., 2000; Miyachi et al., 2004; Sugawara et al., 2010). In the present study, day-to-day coefficients of variation (CVs) for carotid systolic BP and carotid artery diameter were 5.1 ± 1.0% and 3.4 ± 0.5%, respectively.

Elastic tissue properties were assessed using an ultrasound shear wave elastography system (Aixplorer; Supersonic Imagine). Shear wave speed of the carotid arterial wall was obtained with a linear array probe (SL15-4, Supersonic Imagine) in SWE mode (musculoskeletal preset, persistence = off, smoothing = 5). A region of interest (ROI) was positioned on the longitudinal section of the distal common carotid artery, 2-3 cm before the bifurcation, and a video image was recorded for 8 s. Time points corresponding to maximum systolic expansion of the carotid artery and basal (minimum) diastolic relaxation were identified, and three beats of each phase were converted into still images. The value of shear elastic modulus (kPa) in each image was determined as the mean of three points in the near walls at the left, center, and right of the image by off-line manual analysis using image-analysis software (OsiriX MD; Pixmeo SARL, Geneva, Switzerland). The mean of the three beats in each phase was used in subsequent analyses. CV as a measure of reproducibility for shear elastic modulus on the 2 days was 6.0 ± 0.7% and 3.9 ± 1.5% in the systolic and diastolic phases, respectively.

BP, HR, and the systemic PWV parameters were measured using an automated device (VS-1500AE/AN; Fukuda Denshi, Tokyo, Japan) with participants in the supine position, as described previously (Nishiwaki et al., 2017; Nishiwaki et al., 2019). Cuffs to measure pressure waveform were then wrapped around both upper arms and ankles, and heart-ankle PWV (haPWV) and cardio-ankle vascular index (CAVI) were evaluated.

### Statistical analysis

Results are presented as mean ± standard error of the mean (SEM). Changes in parameters were analyzed by two-way (trial × time) repeated-measures ANOVA. When the F value was significant, the Bonferroni method was applied for *post hoc* multiple comparisons. All statistical analyses were performed using Excel Statistic version 4.02 (Bell Curve, Tokyo, Japan). All significance levels were set at 5%.

## Results

Table 2 shows the HR values obtained in each trial. No significant difference in baseline HR was observed between the CON and CS trials. The 5-, 10-, and 15-min HR values were slightly higher during the CS trials than the CON trials, but the difference was not statistically significant. No significant change in any time-course value was observed in either trial.

**TABLE 2:** Heart rate during each trial.

Trial	Baseline			5 min			10 min			15 min		
CON	72	±	2	70	±	2	71	±	2	73	±	3
CS	75	±	3	76	±	3	76	±	3	77	±	3

CON, control trial; CS, cervical stretching trial; Baseline, before each trial (sitting). Data are expressed as mean ± SEM, beats/min. No significant differences or changes were observed.

Table 3 lists the hemodynamic values at baseline and during each trial. Compared with the baseline values, there was no significant change in HR, carotid BP, carotid diameter, and brachial BP in either trial. However, in the CS trial, carotid PP and diastolic diameter of the carotid artery tended to decrease after cervical stretching.

**TABLE 3:** Hemodynamic responses before and after each trial.

Variable		Pre			0 min			15 min			30 min			Interaction
Heart rate, beats/min	CON	62	±	2	57	±	2	58	±	2	58	±	2	F = 0.989
	CS	61	±	3	58	±	2	58	±	2	59	±	2	*p* = 0.401
Carotid systolic BP, mmHg	CON	115	±	4	117	±	3	116	±	4	115	±	3	F = 0.878
	CS	118	±	3	117	±	4	115	±	3	116	±	3	*p* = 0.455
Carotid pulse pressure, mmHg	CON	44	±	3	44	±	2	44	±	3	43	±	3	F = 0.885
	CS	48	±	3	46	±	4	43	±	2	42	±	2	*p* = 0.452
Carotid arterial maximal diameter, mm	CON	6.7	±	0.1	6.6	±	0.1	6.8	±	0.1	6.7	±	0.1	F = 1.291
	CS	6.7	±	0.1	6.6	±	0.1	6.7	±	0.1	6.7	±	0.1	*p* = 0.282
Carotid arterial minimum diameter, mm	CON	6.2	±	0.1	6.0	±	0.1	6.3	±	0.1	6.2	±	0.1	F = 1.224
	CS	6.2	±	0.1	6.1	±	0.1	6.1	±	0.1	6.3	±	0.1	*p* = 0.305
Brachial systolic BP, mmHg	CON	119	±	3	120	±	3	118	±	3	118	±	3	F = 0.041
	CS	118	±	2	119	±	2	119	±	2	117	±	2	*p* = 0.989
Brachial diastolic BP, mmHg	CON	71	±	2	73	±	2	73	±	1	72	±	2	F = 1.748
	CS	71	±	2	71	±	1	72	±	2	73	±	2	*p* = 0.162
Brachial mean BP, mmHg	CON	89	±	2	91	±	2	90	±	2	90	±	2	F = 1.210
	CS	89	±	2	89	±	2	89	±	2	91	±	2	*p* = 0.310
Brachial pulse pressure, mmHg	CON	48	±	2	47	±	2	45	±	2	46	±	2	F = 1.317
	CS	47	±	2	48	±	2	45	±	2	44	±	2	*p* = 0.273

CON, control trial; CS, cervical stretching trial; Pre, before each trial (baseline); BP, blood pressure. Data are expressed as mean ± SEM. No significant differences or changes were observed.

**FIGURE 3:**
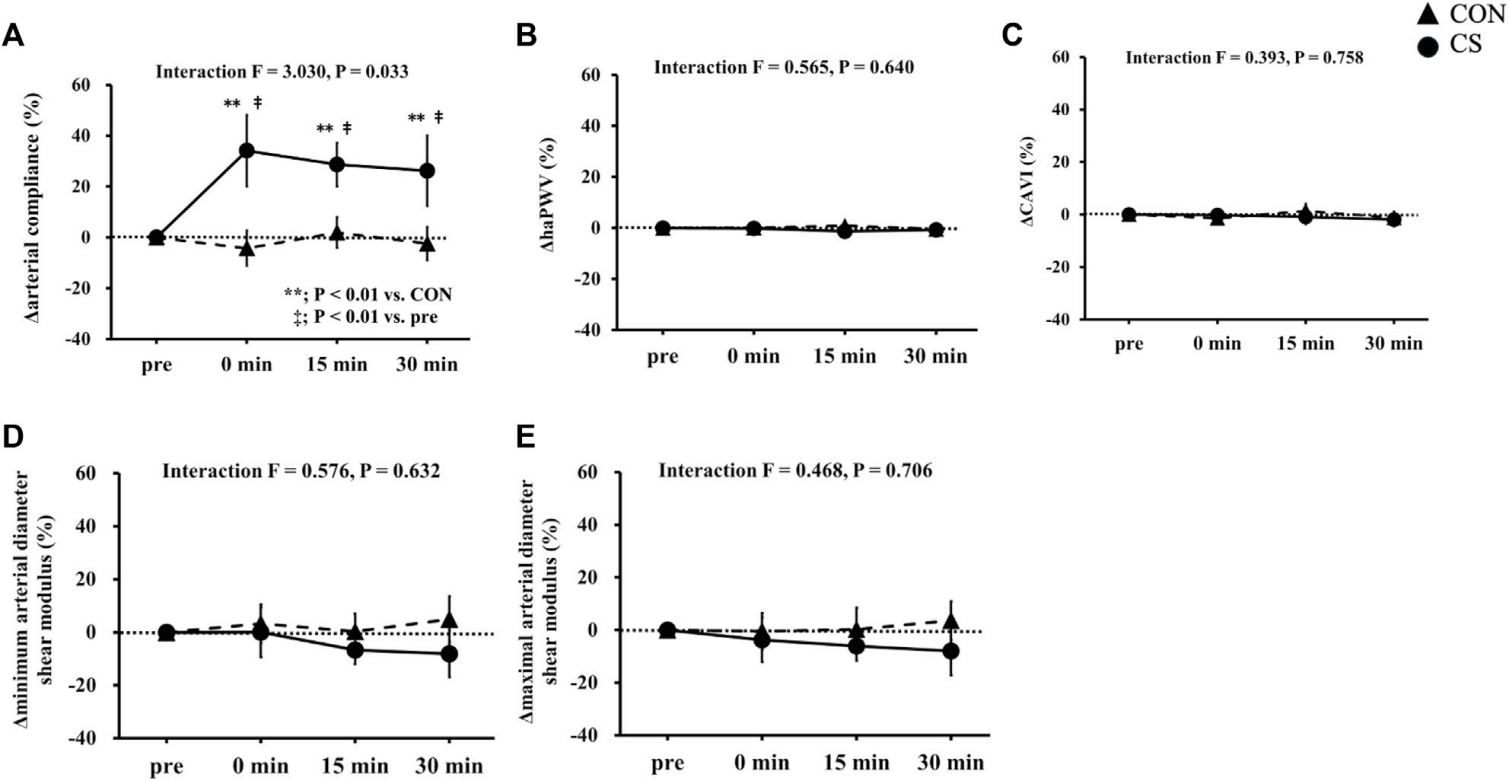
Effects of cervical stretching. Effects on carotid arterial compliance **(A)**, haPWV **(B)**, CAVI **(C)**, minimum arterial diameter shear modulus **(D)**, and maximal arterial diameter shear modulus **(E)**. CON, control sitting trial; CS, cervical stretching trial; haPWV, heart-ankle pulse wave velocity; CAVI, cardio-ankle vascular index. The closed triangles show changes in the CON trial and closed circles show changes in the CS trial. Data are expressed as mean ± SEM. Cervical stretching significantly increased carotid arterial compliance in the CS trial (*p* = 0.033) but did not change elastic tissue properties (*i.e.*, main changes in arterial structural factors) of the carotid artery wall.

Figure 3 shows changes in carotid arterial compliance, systemic arterial stiffness, and shear elastic modulus. Two-way repeated-measures ANOVA revealed significant interaction in carotid arterial compliance from baseline in both absolute (*p* = 0.041) and relative values (*p* = 0.033). Baseline arterial compliance was 0.10 ± 0.01 mm^2^/mmHg and 0.12 ± 0.01 mm^2^/mmHg in the CON and CS trials, respectively, after which the values showed a significant increase immediately, at 15 min, and at 30 min after cervical stretching in the CS trial but not in the CON trial. Baseline haPWV was 604 ± 11 cm/s and 609 ± 8 cm/s in the CON and CS trials, respectively, and baseline CAVI was 5.9 ± 0.1 unit and 6.0 ± 0.1 unit in the CON and CS trials, respectively. Systemic arterial stiffness, assessed by haPWV and CAVI, did not change significantly in either trial. Furthermore, no significant interactions in shear elastic modulus were found in the minimum or maximal phase, in either the absolute values (minimum, *p* = 0.798; maximal, *p* = 0.926) or relative values from baseline (minimum, *p* = 0.632; maximal, *p* = 0.706). In the minimum phase, baseline shear elastic modulus was 47.4 ± 2.9 kPa and 50.6 ± 2.3 kPa in the CON and CS trials, respectively; in the maximal phase, these values were 44.5 ± 2.6 kPa and 48.1 ± 2.2 kPa, respectively. Overall, in both the minimum and maximal phases, the shear elastic modulus of the arterial wall tended to decrease 15 min and 30 min after cervical stretching in the CS trial. However, there was no significant alteration in the shear elastic modulus of the arterial wall after stretching in either phase, in either trial.

## Discussion

To the best of our knowledge, this is the first study to evaluate the effects of cervical stretching exercise on carotid artery compliance and tissue elastic properties of the carotid arterial wall in humans. The main findings were that acute supervised cervical stretching (*i.e.*, in the CS trial) increased carotid arterial compliance, but was not observed in the CON trial; and that cervical stretching (*i.e.*, in the CS trial) induced no statistically significant reduction in shear elastic modulus.

Several recent studies have assessed the tissue elastic properties of the arterial wall using the SWE system, with quantitative methods attracting increasing attention (Ramnarine et al., 2014; Garrard et al., 2015; Pruijssen et al., 2020; Thomas et al., 2020; Al-Mutairi et al., 2022). The measurement validity of these methods has been verified in phantoms by mechanical testing, *ex vivo*, and systematic review approaches (Maksuti et al., 2016; Widman et al., 2016; Pruijssen et al., 2020). In particular, a systematic review demonstrated that SWE could detect statistically significant elasticity differences in patient/subject characteristics and could distinguish different plaque types with good reproducibility (Pruijssen et al., 2020). A previous study has reported that shear elastic modulus values of the carotid arterial wall ranged from 34 kPa to 87 kPa in healthy control participants (Al-Mutairi et al., 2022), whereas another reported an average value of 58 kPa in participants with a mean age of 40 ± 10 years (Alyami and Almutairi, 2022). In the present study, baseline average values of shear elastic modulus in each phase ranged from 44.5 ± 2.6 kPa to 50.6 ± 2.3 kPa, which approximate the measurement levels and ranges reported previously. Accordingly, the present tissue elastic property values quantified by the SWE system have high validity.

Acute stretching of a local body part is well known to elicit an increase in arterial compliance and a reduction in arterial stiffness in that body part (Yamato et al., 2017; Higaki et al., 2021; Ikebe et al., 2022b). Our results are consistent with those of previous studies and indicate that acute cervical stretching increased carotid arterial distensibility without reducing systemic arterial stiffness (haPWV and CAVI). Arterial compliance alterations are generally considered to result from arterial structural factors, arterial functional factors, or a combination of the two. Changes in arterial functional factors are widely known, but it is unclear whether changes in arterial structural factors are induced by stretching and exercise in humans. Our study thus evaluated the effects of cervical stretching on the tissue elastic properties of the arterial wall as the change in arterial structural factors, using the noninvasive SWE method. However, we did not detect any significant change in shear elastic modulus in either cardiac phase. In studies that have conducted mechanical testing in phantoms or *ex vivo* testing (Maksuti et al., 2016; Widman et al., 2016), the values of shear elastic modulus have been taken to indicate tissue elastic properties and are considered to mainly reflect the status of arterial structural factors. The SWE method can also detect stretching-induced changes in skeletal muscle stiffness (Hirata et al., 2017; Nakamura et al., 2017; Taljanovic et al., 2017). Thus, our results at least indicate that present stretching did not induce such major tissue elastic property changes as quantifiable by the SWE method, meaning that the changes are less important in an increase in arterial compliance. Therefore, carotid artery compliance increased significantly in the present study, but the findings indicate that acute cervical stretching could not reduce the elastic tissue properties of the carotid artery wall in young participants. These results strongly suggest that changes in structural factors have little relation to stretching-induced acute increases in arterial compliance.

We can only speculate on the physiological mechanisms by which cervical stretching increases arterial compliance, which may be as follows. First, because there was no alteration in the elastic tissue properties of the arterial wall after stretching, it seems unlikely that changes in arterial structural factors are strongly involved in the increase in compliance. Indeed, the findings of animal studies indicate that arterial structural changes (*i.e.*, in the composition of elastin and collagen) are less likely to occur in a relatively short period (Tanaka et al., 2000; Nosaka et al., 2003; Maeda et al., 2005). Second, in terms of arterial functional factors, previous studies have reported that stretching induces blood flow and shear rate during the relaxation period (Yamato et al., 2021), and that this stimulus can cause a reduction in arterial stiffness (*i.e.*, an increase in arterial compliance) after stretching (Hotta et al., 2013; Higaki et al., 2021; Yamato et al., 2021; Hotta et al., 2018). In our experimental data, an increasing tendency of shear stress immediately after cervical stretching was observed (CON vs. CS: 1.4 ± 5.9% vs. 10.7 ± 5.1%). These data suggest that changes in blood flow and shear rate during the relaxation period of stretching exercises contribute to increased arterial compliance. Furthermore, it is possible that sympathetic vasoconstrictor tone reduces with exercise (stretching), thereby increasing arterial compliance (Sugawara et al., 2009). In support of this possibility, recent acute studies have indicated that comedy-induced mirthful laughter increases arterial compliance (Sugawara et al., 2010), whereas mental stress increases arterial stiffness (*i.e.*, decreases arterial compliance) (Kume et al., 2020; Kume et al., 2021). Thus, stretching stimulus could affect sympathetic vasoconstrictor tone. However, since changes in arterial function (*i.e.*, compliance and stiffness) were observed only in the carotid artery, the compliance alternations seem unlikely to result from sympathetic tone-induced systemic effects. Alternatively, stretching is well known to induce quantifiable changes in skeletal muscle stiffness (Hirata et al., 2017; Nakamura et al., 2017). Thus, the reduction in internal pressure due to changes in skeletal muscle stiffness may easily have induced arterial distension during the systolic period. For this reason, we attempted to quantify tissue elastic properties in the cervical muscle on a trial basis. There was a slight and consistent decreasing tendency from the baseline values (CON, 0 min systolic delta 2.4 ± 6.4%, diastolic delta 5.2 ± 6.2%; 15 min systolic delta 11.0 ± 8.7%, diastolic delta 7.1 ± 8.0%; CS, 0 min systolic delta 0.1 ± 6.1%, diastolic delta −1.7 ± 5.7%; 15 min systolic delta 0.4 ± 6.8%, diastolic delta 0.1 ± 6.8%). However, no significant differences or changes in tissue elastic properties were detected in the cervical muscle. Thus, a stretching-induced reduction in internal pressure due to changes in skeletal muscle stiffness seems unlikely to contribute to the increase in arterial compliance. Therefore, these findings suggest that acute alternations in arterial compliance might contribute to changes in arterial functional factors rather than changes in arterial structural factors. However, further studies are required to clarify the physiological mechanisms in more detail.

This study has some limitations. This study was performed using an acute design and approach as an initial pilot study. Thus, the participants were young, healthy adults with no chronic disease, and had high initial values of central arterial distensibility (Tanaka et al., 2000). The elastic tissue properties of the carotid artery wall might not change readily, especially in young individuals. Thus, our results are specific to young, healthy participants, and additional investigations using different protocols and study populations might uncover important new insights into the relationship between increased arterial compliance and changes in arterial structural factors. Further investigations are thus required to elucidate these points.

In conclusion, acute cervical stretching significantly increased carotid artery compliance, but no reduction in elastic tissue properties (*i.e.*, changes in arterial structural factors) of the carotid artery wall was found in young participants. These results strongly suggest that changes in structural factors have little relation to stretching-induced acute increases in arterial compliance.

## Data Availability

The raw data supporting the conclusion of this article will be made available by the authors, without undue reservation.
